# Influence of body mass index on Benign Prostatic Hyperplasia-related complications in patients undergoing prostatectomy

**DOI:** 10.1186/2193-1801-2-537

**Published:** 2013-10-17

**Authors:** Hisham A Mosli, Hala H Mosli

**Affiliations:** Department of Urology, King Abdulaziz University, PO Box 80215, Jeddah, 21589 Saudi Arabia; Department of Medicine (Endocrinology), King Abdulaziz University, PO Box 80215, Jeddah, 21589 Saudi Arabia

## Abstract

**Objectives:**

To examine the link between increased body mass index and benign prostatic hyperplasia (BPH) related complications, namely: acute urinary retention (AUR), Bladder stones and Bladder diverticula.

**Material and methods:**

Patients: We reviewed the medical records of BPH patients who underwent prostatectomy over three years period from 2010–2012. Prostatectomy was either done in the gold standard transurethral resection of the prostate (TURP) or using green light laser selective photo-vaporization (PVP). Age, PSA, Prostate Volume (PV) as measured by ultrasonography, patients’ weight & height, BPH related complications mainly AUR, bladder stones, and bladder diverticula were all taken in account. The BMI was calculated as weight in kg divided by square height in meters. The BMI was divided in 4 categories according to WHO classification: underweight if BMI ≤18.50 kg/m2; normal weight if BMI = 18.50-24.9 kg/m2; overweight: BMI ≥25 kg/m2; and obese BMI if ≥30 kg/m2. Statistical analysis: done using the SPSS package version 16. Chi-square test was used for comparison between groups where p-value was considered significant if <0.05 and ANOVA test was used for comparison between multiple variables.

**Results:**

197 patients were included in this study, of those 95(48%) underwent TURP and 102(52%) underwent PVP. The two groups were found to be similar in mean age and PSA, and significantly different in prostate volume and operating room (O.R.) time.

**Conclusions:**

Although the mean weight for patients undergoing prostatectomy in this study was in the overweight range, yet only 59/197(30%) patients with increased BMI presented with AUR. In this particular cohort of patients undergoing prostatectomy there was no significant differences in the development of AUR, bladder stone and diverticulum formation between patients with increased BMI (overweight and obese) and those with normal BMI. Further studies are recommended to explore the influence of increased BMI on BPH-related complications.

## Introduction

Obesity has recently been declared to be associated with several urological diseases (Hammarsten et al. 1998). Central obesity and lack of physical exercise were placed among the top risk factors for the development of BPH (Hammarsten et al. 1998; Hammarsten & Högstedt 1999; Hammarsten & Högstedt 2001; Hammarsten & Högstedt 2002; Hammarsten et al. 2009). It has been shown in the placebo arm of Reduce® study that the prostatic growth is accelerated in overweight and obese patients while in the treatment arm the response to treatment with 5-alpha reductase enzyme inhibitors was attenuated in those patients (Kaplan & Wilson 2007; Muller et al. 2012). It is therefore assumed that increased BMI is linked to increased prostate sizes in patients undergoing surgical treatment namely prostatectomy and when examining the BPH-related complications such as retention of urine (AUR), bladder stone and diverticulum formation, we anticipated that these complications were higher in overweight and obese patients than in normal weight patients undergoing prostatectomy.

## Objectives

The objective of this study was to examine the link between increased BMI and BPH related complications. Those complications examined were: AUR, Bladder stones and Bladder diverticula.

## Material and methods

### Patients

We reviewed the medical records of BPH patients who underwent prostatectomy over three years period from 2010–2012. Prostatectomy was either done in the gold standard transurethral resection of the prostate (TURP) or using green light laser selective photo-vaporization (PVP). Age, PSA, Prostate Volume (PV) as measured by ultrasonography, patients’ weight & height, BPH related complications mainly AUR, bladder stones, and bladder diverticulum were all taken in account. The BMI was calculated as weight in kg ÷ square height in meter. The BMI was divided in 4 categories according to WHO classification: underweight if BMI ≤18.50 kg/m2; normal weight if BMI = 18.50-24.9 kg/m2; overweight: BMI ≥25 kg/m2; and obese BMI if ≥30 kg/m2. All participants signed an informed consent prior to inclusion in the study. The study was approved by the Institution’s Unit of Biomedical Ethics- Research Committee.

Statistical Analysis: Was done using the SPSS package version 16. Chi-square test was used for comparison between groups where p-value was considered significant if <0.05 and ANOVA test was used for comparison between multiple variables.

## Results

197 patients were included in this study, of those 95(48%) underwent TURP and 102(52%) underwent PVP. The two groups were found to be matched in mean age and PSA, and significantly different in prostate volume and operating room (O.R.) time. The results are summarized in Tables 1, 2, 3, 4 and 5. Further analysis showed that of the 95 patients undergoing TURP, 38 (40%, 19.2% of total study population) were in the underweight-normal BMI group, whereas 67 (60%, 28.8% of total study population) were in the overweight-obese BMI group. Of the 102 patients undergoing PVP, BMI data was available for 77 patients. Analysis of this data showed that 34 (44.15%) were in the underweight-normal BMI group, and 43 (55.85%) were in the overweight-obese BMI group.

**Table 1:** **Patients undergoing TURP, PVP grouped by BMI**

Procedure		BMI				Total	X2
		Underweight	Normal	Overweight	Obese		P-vale
TURP	Count	1	37	41	16	95	10.447
	%	1.1%	38.9%	43.2%	16.8%	100.0%	
PVP	Count	6	28	21	22	77	
	%	7.8%	36.4%	27.3%	28.6%	100.0%	0.015*
Total	Count	7	65	62	38	172	
	%	4.1%	37.8%	36.0%	22.1%	100.0%	

*significant using chi-square test at 0.05 level.

**Table 2:** **Showing the similarities and differences between the study’s prostatectomy groups**

	Group	N	Mean	Std. Deviation	P-value
Age	TURP	95	68.36	8.67	0.020
	PVP	121	71.24	9.07	
O.R. time	TURP	95	86.48	37.56	<0.0001
	PVP	79	61.12	40.38	
Prostate volume	TURP	95	60.95	33.32	<0.0001
	PVP	101	46.93	14.30	
PSA	TURP	94	9.17	39.00	.262
	PVP	100	4.71	6.58	

*Abbreviations: TURP* transurethral resection of the prostate, *PVP* photo-selective vaporization of the prostate, *O.R.* operative room, *PSA* prostate specific antigen.

**Table 3:** **Shows the BMI categories of the study population**

		Frequency	Percent
	Underweight	7	4.06
	Normal	65	37.79
	Overweight	62	36.04
	Obese	38	22.09
	Sub-total	172	87.30
Data	Not available	25	12.69
Total		197	100.0

**Table 4:** **Showing the data for the whole cohort**

Age	Minimum 51	Mean 68.3684 years
	Maximum 90 years	standard deviation 8.67
PSA	Minimum 00.00	Mean 6.87 ng/ml
	Maximum 373.00	standard deviation 27.57
PV	Minimum 15	Mean 54.11
	Maximum 200 c.c.	standard deviation 26.52 c.c.
BMI	Minimum 16.8	Mean 26.39 kg/m^2^ overweight>25 kg/m^2^
	Maximum 38.28	standard deviation 4.39
AUR	74 /197	37.6%
Bladder stones	17/180	8.6%
Diverticulum	3/194	1.5%

*Abbreviations: PSA* prostate specific antigen, *PV* prostate volume, *BMI* body mass index, *AUR* acute retention of urine.

**Table 5:** **Showing influence of BMI on BPH-related complications**

Group			DIVERTICULUM		Total	RETENTION		Total	BLADDER STONE		Total
			Absence	Present		Absence	Present		Absence	Present	
TURP	Normal	Count	37	1*	38	22	16**	38	33	5***	38
		%	97.40%	2.60%	100.00%	57.90%	42.10%	100.00%	86.80%	13.20%	100.00%
	Overweight & Obese	Count	55	2*	57	33	24**	57	47	10***	57
		%	96.50%	3.50%	100.00%	57.90%	42.10%	100.00%	82.50%	17.50%	100.00%
		Count	92	3	95	55	40	95	80	15	95
		%	96.80%	3.20%	100.00%	57.90%	42.10%	100.00%	84.20%	15.80%	100.00%
PVP	Normal	Count	28		28	15	13****	28	28	0*****	28
		%	100.00%	-	100.00%	53.60%	46.40%	100.00%	100.00%	0.00%	100.00%
	Overweight & Obese	Count	37		37	31	6****	37	36	1*****	37
		%	100.00%	-	100.00%	83.80%	16.20%	100.00%	97.30%	2.70%	100.00%
		Count	65		65	46	19	65	64	1	65
		%	100.00%	-	100.00%	70.80%	29.20%	100.00%	98.50%	1.50%	100.00%

*Abbreviations: TURP=Transurethral resection of the prostate, PVP= photo-selective vaporization of the prostate.*

* P= 0.50.

** P=0.66.

*** P=0.50.

**** P=0.45.

***** P= 0.06.

## Discussion

Evidence elicited from the current study did not demonstrate an increase in the risk of developing BPH-related complications with an increased BMI. No significant differences were noted in the development of AUR, bladder stone and diverticulum formation; between patients with increased BMI (overweight and obese) and those with normal BMI. A relationship between higher BMIs and elevated PSA, poorer uroflow, increased incidence of retention, and larger prostate volume were expected to be seen, however our study demonstrated a relationship that was NOT statistically significant.

The metabolic syndrome is common in Arabian Gulf countries especially Saudi Arabia. It comprises a number of disorders—including insulin resistance, hypertension and central abdominal obesity—that all act as risk factors for cardiovascular diseases. Accumulating evidence now exists to link urological diseases to the metabolic syndrome (Hammarsten et al. 1998). Most established aspects of the metabolic syndrome are primarily linked to benign prostatic hyperplasia (BPH) and prostate cancer (Hammarsten et al. 1998; Hammarsten & Högstedt 1999; Hammarsten & Högstedt 2001; Hammarsten & Högstedt 2002; Hammarsten et al. 2009). Fasting plasma insulin, in particular, was linked to BPH and all subtypes of prostate cancer namely: incidental, aggressive and lethal prostate cancer (Hammarsten & Högstedt 2002). Medical treatment is thought to be less efficacious in obese patients with symptomatic BPH than normal weight patients (Lee et al. 2011). Overall, the results of studies on urological aspects of the metabolic syndrome seem to indicate that BPH and prostate cancer are recently considered as two aspects of the metabolic syndrome, and that an increased insulin level is a common underlying aberration that promotes both BPH and clinical prostate cancer (Nandeesha et al. 2006).

Affluence associated with prosperity in wealthy countries has resulted in some serious health problems due to overindulgence in the consumption of high calorie foods and sugar sweetened beverages, and intake of excessive amounts of fast and fatty food. Obesity follows with all its sequences, especially when living a sedentary life and lack of protective regular physical activities against cardiovascular diseases (Guo et al. 2005).

**Figure 1:**
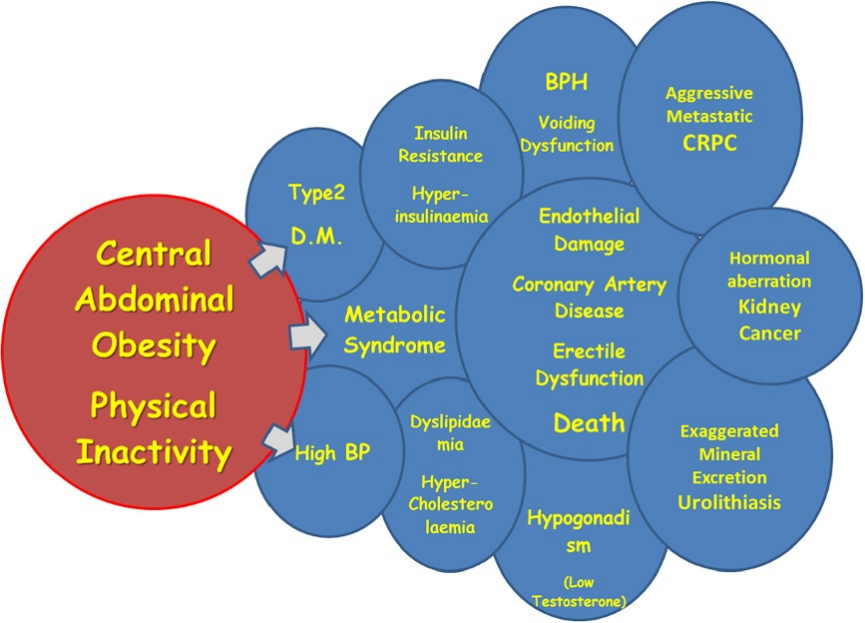
**Illustrating the links between Obesity and affected health parameters.**

The main concern with the metabolic syndrome is the cardiovascular diseases, mainly coronary artery disease (CAD) as this is a leading cause of death. Furthermore, an association between benign prostatic hyperplasia and primary hypertension was reported (Guo et al. 2005). The relationships between body mass index and lower urinary tract symptoms (LUTS) were also reported. The links of central obesity and lack of physical exercise to some medical conditions are all illustrated in Figure 1.

Obesity is measured by several methods, but for practical purposes and simplicity, it is represented in clinical urology by WC or BMI (Hammarsten & Högstedt 1999). Recent data suggested a relationship between the WC and health parameters, mainly diabetes, hypertension, prostate volume (PV), voiding and sexual dysfunction (Hammarsten & Högstedt 1999).Diabetes mellitus has been extensively discussed as a risk factor for many urological disorders, mainly voiding and sexual dysfunction (Ochiai et al. 2005; Li et al. 2005). Furthermore, there is evidence that type 2 diabetes mellitus is associated to, linked to, or even a direct sequel of obesity through the development of insulin resistance (Parsons et al. 2006).The resultant hyperinsulinemia (Parsons et al. 2009; Ozden et al. 2007; Keto et al. 2011; De Nunzio et al. 2012) plays a major role in the pathophysiological changes that occur in the genitourinary system and throughout the whole human body as shown in Figure 1.

Evidence from a large prospective study indicates that a progressive increase in the BMI is associated with progressive increase in PV and attenuated response to treatment with 5-alpha reductase inhibitors (Kaplan & Wilson 2007; Muller et al. 2012; Lee et al. 2011; Roehrborn et al. 2006). In our opinion this finding has an important relevant therapeutic implication in the medical treatment of obese men with BPH. This also warrants further research studies on the relationship between the degree of obesity and unresponsiveness to medical therapy and the development of complications of BPH such as retention of urine, bladder stones and diverticula formation.

## Conclusions

Although the mean weight for patients undergoing prostatectomy in this study was in the overweight range, yet only 59/197(30%) patients with increased BMI presented with AUR. In this particular cohort of patients undergoing prostatectomy there was no significant differences in the development of AUR, bladder stone and diverticulum formation between patients with increased BMI (overweight and obese) and those with normal BMI. Further studies are recommended to explore the influence of increased BMI on BPH-related complications. Subsequently, active life style and weight reduction can be discussed with patients with symptomatic BPH within the context of benefit not only in improving LUTS, and slowing the growth of the prostate but also to improve response to medical therapy and prevent BPH related complications.
